# High Prevalence and Burden of Physical and Psychological Symptoms in a Chronic Obstructive Pulmonary Disease Population in Primary Care Settings in South Africa

**DOI:** 10.2147/COPD.S395834

**Published:** 2023-08-02

**Authors:** Kennedy B Nkhoma, Lindsay Farrant, Olona Mzimkulu, Joy Hunter, Irene Higginson, Wei Gao, Matthew Maddocks, Liz Gwyther, Richard Harding

**Affiliations:** 1 Cicely Saunders Institute of Palliative Care Policy and Rehabilitation, King’s College London, London, UK; 2 Division of Interdisciplinary Palliative Care & Medicine, Department of Public Health and Family Medicine, Faculty of Health Sciences, University of Cape Town, Cape Town, South Africa

**Keywords:** chronic lung disease, chronic obstructive pulmonary disease, symptom burden, symptom distress, symptom prevalence

## Abstract

**Background:**

Many deaths globally are attributable to non-communicable disease, and four-fifths of these deaths are in low- and middle-income countries. Globally, COPD is currently the third leading cause of mortality.

**Research Question:**

1) To determine the prevalence and burden of symptoms and concerns, and 2) determine predicting factors of symptom burden among patients with COPD.

**Methods:**

A cross-sectional survey was conducted at eight primary care sites in Western Cape. We collected socio-demographic data (age, gender, smoking status, number of missed doses of prescribed medication in the last seven days) and clinical data (PEF and KPS). The Memorial Symptom Assessment Scale (MSAS), the Medical Outcomes Study, Social Support Survey (MOS-SSS), the London Chest Activity of Daily Living Scale (LCADLS) and the COPD Assessment Test (CAT) (impairment on person’s life) were administered to patients. We conducted ordered logistic regression analysis to assess factors associated with the burden of symptoms. MSAS subscales: 1) Global symptom distress index, 2) physical symptom distress and 3) psychological symptom distress were dependent outcomes. We constructed three ordinal logistic regression models for each of the three subscales. Covariates were MOS-SSS, LCADLS, CAT, demographic and clinical variables.

**Results:**

We recruited n=387 patients, mean age 59.5 years, 53.0% female. In multivariate analysis, each of the three models (ie, global, psychological and physical symptom distress) was positively associated with impairment on person’s life p<0.001, difficulty to perform activities of daily living p<0.001, and low social support p<0.001. Old age was associated with lower global symptom distress (p=0.004), psychological and (0.014) physical distress (0.005). Missing 1 or more doses of medication was associated with higher levels of global (0.004) and physical (0.005) symptom distress.

**Interpretation:**

The high prevalence and burden of physical and psychological symptoms provides strong evidence of the need for integrating person-centred assessment and management of symptoms in primary care settings.

## Background

The majority of serious health-related suffering is attributable to non-communicable diseases (NCD) and is increasing most rapidly in low- and middle-income countries (LMICs)^1^ and four-fifths of these deaths are in LMICs.^2^

The WHO’s global burden of disease data estimate that in 2019 212.3 million adults were living with COPD.^3^ It has been projected that by 2030 NCDs will be the most common causes of mortality in LMICs,^4^ due to a combination of increasing and aging populations. More than 90% of COPD deaths occur in LMICs.^5^,^6^

WHO estimated that in South Africa the burden from NCD is two to three times higher than in developed countries,^4^ furthermore, National and Western Cape Province data show a high mortality from diseases of the circulatory and respiratory systems.^7^,^8^

COPD patients often experience exacerbations of their disease which require hospitalisation. After the first hospitalisation, half of the patients die within 3.6 years.^9^ COPD has unpredictable course, with sudden and life-threatening exacerbations, often leading to ad hoc decisions about how to proceed. Evidence from a systematic review on utilisation and costs of services among advanced COPD patients found increased hospitalisations, intensive care unit stays, primary care consultations and medication prescription, as well as a lack of palliative care services.^10^ COPD can lead to greater financial hardship,^11^ this has great implications for patients and families in LMIC.

The focus of a health system’s strengthening response to increasing prevalence and mortality from NCDs should be on primary care to improve outcomes and reduce inequity.^12^,^13^ Primary care utilisation for chronic disease management can improve patient outcomes and reduce costs,^14^ providing holistic person-centred care to reduce secondary and tertiary care use.

It is important to note the differing aetiology and pathogenesis of COPD in countries such as South Africa, compared to more developed countries. The percentage of patients developing COPD from tobacco smoking is lower in South Africa than in the developed world, although the rates of smoking are not the same across the South African population.^15^ Although tobacco smoking is a risk factor for COPD in LMICs, between a third to a fifth of cases in LMICs occur in people who have never smoked.^16–18^

The other aetiological factors in South Africa are pulmonary tuberculosis, occupational dust exposure, biomass fuel smoke exposure for cooking and heating,^19^ other forms of air pollution, respiratory infections in childhood and HIV, making this a diverse spectrum of disease which may be more accurately described as chronic lung disease (CLD).^15^ Chronic lung disease (CLD) is a type of disorder that affects the lungs and other parts of the respiratory system. CLD develops slowly and worsens over time. Types of chronic lung disease include asthma, chronic obstructive pulmonary disease (COPD), pulmonary fibrosis, asbestosis, pneumonitis, and other lung conditions. In this paper, we will use COPD and not CLD to describe our sample. In Africa, COPD has a prevalence rate of around 13.4%, and 20% in South Africa (SA).^20^,^21^

A systematic review of patient and caregiver views on palliative and end-of-life care reported multidimensional symptoms and concerns.^8^ Most of these patients experience physical symptoms such as pain, breathlessness and may require support with psychosocial or spiritual problems as their diseases progress.^22^ However, this evidence has been collected in high-income countries. In sub-Saharan Africa, the burden of pain and symptoms in HIV, heart failure and cancer patients are associated with poorer quality of life and psychological distress and places a huge burden on patients and (largely female) family caregivers.^23–27^ This study aimed to measure the seven-day period prevalence of physical and psychological symptoms among COPD patients, and to identify predictors of symptom burden.

## Methods

We undertook a cross-sectional study using self-reported data with file extraction and observer ratings. We included adults (at least 18 years of age) with a documented COPD diagnosis. Participants were recruited at eight primary care facilities (two primary care district hospitals, three 24hr Community health centres with Emergency units, and three 8hr Community Health Centres with emergency rooms) in Cape Town, South Africa.

These facilities offer medical outpatient and emergency care services for COPD patients.

In South Africa, primary care occurs in the context of primary health care delivery through the district health services.^28^ Primary care is largely provided by clinical nurse practitioners,^29^ but in the metropolitan area where this study was conducted, doctors are also available at all times of operation. Primary care is provided in clinics that operate for 8 hours and health centres that operate over 24 hours. At each model, COPD patients can access emergency care for nebulisation and oxygen when experiencing severe breathlessness.

Ethical approval was obtained from the King’s College London (HR-17/18-5766) and University of Cape Town (HREC REF 211/2018). This study complies with the Declaration of Helsinki.

Potential participants were identified by clinic staff, and then study-specific research (fluent in local languages) assistants read the information sheet aloud prior to collecting written consent.

### Sample Size Calculation

We calculate a sample size of n=385 to estimate the prevalence of symptoms in an unknown population size with 5% precision and 95% confidence and estimated 50% prevalence. This enabled entry of 10 planned variables in regression analysis.

### Data Collection

Research assistants extracted clinical data patient records which included COPD diagnosis, number of years since diagnosis, Global Initiative for Chronic Obstructive Lung Disease (GOLD) stage, current treatment, medication adherence, number of hospitalisations in previous 12 months, referral patterns and number of referrals in last 12 months, and co-morbidity. The research assistant then measured peak expiratory flow (PEF) and height. The research assistant read aloud self-report questionnaires and recorded participants’ responses. Data were collected after clinical appointments.

Measures used were as follows:

1) Demographic data (age, sex, first language, education level, living arrangements) and socioeconomic status (see Supplementary File 1).

2) The Memorial Symptom Assessment Scale-Short Form^30^,^31^ (MSAS-SF). The MSAS-SF is an abbreviated version of the MSAS, a questionnaire validated in several different populations including patient with heart failure.^32^,^33^ The MSAS-SF enables a multidimensional assessment of symptoms. MSAS-SF measures the prevalence and burden of physical and psychological symptoms in the past seven days.^31^ MSAS-SF has three subscale indices of physical symptom distress (MSAS-PHYS), psychological symptom distress (MSAS-PSYCH) and global distress index (MSAS-GDI), each has a score range of 0–4. MSAS is applicable in a wide range of disease conditions and enables comparison between conditions previously reported using this measure in sub-Saharan Africa (heart failure, HIV and cancer).^27^,^34^,^35^ The global distress index (GDI) consists of four psychologic symptoms: feeling sad, worrying, feeling irritable, and feeling nervous, and six physical symptoms: lack of energy, pain, lack of appetite, feeling drowsy, constipation, and dry mouth. Possible scores range from 0 to 40. The physical symptom distress score (PHYS) includes 12 prevalent physical symptoms (lack of energy, pain, lack of appetite, feeling drowsy, constipation, dry mouth, nausea, vomiting, change in taste, weight loss, feeling bloated, and dizziness). Possible scores range from 0 to 48. The psychologic symptom distress score (PSYCH) includes six psychologic symptoms (worrying, feeling sad, feeling nervous, difficulty sleeping, feeling irritable, and difficulty concentrating). Scores range from 0 to 24.^30^,^31^

3) The Medical Outcomes Study (MOS) Social Support Survey (SSS).^36^ MOS-SSS measures the levels and type of social support. The questionnaire has 19 items that are further divided into 4 subscales, namely emotional/informational supports, tangible support: affectionate support and positive social support, and one additional global item. The tool has been used in several heart failure studies and also in studies conducted in South Africa.^37–39^ MOS-SSS has a well-established reliability and stability (α > 0.91). Scores range from 19 to 95 with higher scores representing better outcomes.

4) The London Chest Activity of Daily Living Questionnaire was used to measure the severity of breathlessness on exertion over the previous 24 hours.^40^ This standardized 15-item questionnaire assesses routine activities of daily living on a Likert scale of 0 (best) to 5 (worst) outcomes.

5) The COPD Assessment Test (CAT) is an 8-item unidimensional measure of health status impairment in COPD^41^,^42^ that scores between 0 (best) to 40 (worst).

6) The Karnofsky Performance Scale Index,^43^ to assess the functional impairment or patients overall performance status, rated on a scale of 0–100, with 0 corresponding to no physical function (death) and 100 corresponding to maximum independent functioning or no evidence of disease.^43^

### Data Analysis

Data were entered were managed using REDCap (Research electronic data capture) tools hosted at the University of Cape Town.^44^,^45^ Data was then imported to Stata version 15 for cleaning and analysis.^46^

We conducted a descriptive analysis of demographic and clinical variables (see Table 1). Descriptive analysis of MSAS-SF symptom burden and prevalence was also conducted. Following calculation of prevalence for each symptom, burden for each prevalent symptom was calculated as: 0.8 “no distress at all”, 1.6 “a little bit”, 2.4 “somewhat”, 3.2 “quite a bit” and 4.0 “very much”. Calculation of psychological symptom prevalence was followed by calculation of burden: 1 “rarely” 2 “occasionally” 3 “frequently” and 4 “almost constantly”. The most distressing symptoms were identified: these were symptoms scored using the worst two categories of burden with at least 60% in the worst categories (ie, causes “quite a bit” or “very much” distress for physical symptoms and “frequently” or “almost constantly” for psychological symptoms). Participants under these two worst categories were regarded as experiencing high distress (see Table 2).

**Table 1:** Demographic and Clinical Characteristics n=387

**Demographic Characteristics**	
Gender	
Male	182 (47.03%)
Female	205 (52.97%)
Mean age (SD)	59.52 (10.1)
Median age (IQR)	58 (Q1-Q3=53–67)
Mean years since diagnosis (SD) (169 missing data)	8.77 (9.15)
Median years since diagnosis (IQR)	5 (Q-Q3=3–40)
First language	
Afrikaans	213 (55.04%)
English	78 (20.16%)
Xhosa	92 (23.77%)
Other	4 (1.03%)
Facility	
False Bay Hospital	41 (10.59)
Wesfleur Hospital	40 (10.34)
Delft CHC	105 (27.13)
Gugulethu CHC	44 (11.37)
Eerste River Hospital	21 (5.43)
Nyanga CDC	38 (9.82)
Heideveld CDC	77 (19.9)
Grassy Park CDC	21 (5.43)
Education	
None/Primary school	207 (53.49%)
High school and above	180 (46.51%)
Income (3 missing data)	
Employed	45 (11.72%)
Unemployed	64 (16.67%)
Disability grant/pension	275 (71.61%)
**Behavioural characteristics**	
Smoking status (2 missing data)	
Yes	174 (45.19%)
No	147 (54.81%)
Mean (SD) years since diagnosis	8.77 (9.16)
COPD GOLD Stage (337 missing data)	
Stage 1	4 (8%)
Stage 2	29 (58%)
Stage 3	13 (26%)
Stage 4	4 (8%)
Missed doses (2 missing data)	
None	318 (82.6%)
One or more doses	67 (17.4%)
Living arrangement (1 missing data)	
Lives alone	36 (9.33%)
Does not live alone	350 (90.67%)
Living situation (3 missing data)	
Formal with sanitation inside	317 (82.55%)
Formal with sanitation outside/informal	67 (17.45%)
Number of hospitalisations last 12 months (12 missing data)	
No hospitalisation	176 (46.93%)
One or more hospitalisation/s	199 (53.07%)
Referral to other services last 12 months (133 missing data)	
Respiratory medicine	73 (18.86%)
Physiotherapy	9 (2.33%)
Counselling services	2 (0.52%)
Social worker	63 (16.28%)
Mean (SD) PEF for males (1 missing data)	176.67 (92.2)
Median (IQR)	160 (Q1-Q3=110–230)
Mean (SD) PEF for females (5 missing data)	171.1 (76.83)
Median (IQR)	170 (Q1-Q3=110–225)
Co-morbidity	
None	298 (77%)
One or more co-morbid	89 (23%)
Mean height in cm (SD) (266 missing data)	
Male	167.29 (11.48)
Female	154.96 (8.16)
Mean KPS (SD) (3 missing data)	72.81 (14.27)
Median KPS (IQR)	80 (Q1-Q3=70–80)
KPS≤70%	184 (47.92)
KPS ≥ 80%	200 (52.08)
GDI (MSAS-GDI) mean (SD) (0–40)^a^	16.69 (9.12)
Physical symptom subscale (MSAS-PHYS) mean (SD) (0–48)^a^	15.86 (9.56)
Psychological symptom subscale (MSAS PSYCH) mean (SD) (0–24)^a^	10.05 (6.39)
COPD Assessment test score mean (SD) (0–40)^b^	26.39 (7.57)
LCADL total score mean (SD) (0–75)^c^	28.73 (12.56)
Social support (MOS-social support survey) mean (SD) (19–95)^d^	74.88 (20.88)

**Notes**: ^a^Low scores better outcomes. ^b^Low scores better outcomes. ^c^Low scores better outcomes. ^d^Higher scores better outcomes.

**Abbreviations**: CDC, Community Day centre; CHC, Community health centre; GDI, Global Distress Index; GOLD, Global Initiative for Obstructive Lung Disease; KPS, Karnofsky Performance Status; LCADL, London Chest Activity of Daily Living Scale; MOS, Medical Outcomes Study; MSAS, Memorial Symptom Assessment Scale; PEF, Peak Expiratory Flow; PSYCH, Psychological; PHYS, Physical.

**Table 2:** Prevalence and Burden of Physical and Psychological Symptoms (n=387)

**Physical Symptoms**	**Prevalence**	**Not Present**	**Burden of Prevalent Symptoms n (%)**				
			**Not at All (1)**	**A Little Bit (2)**	**Somewhat (3)**	**Quite a Bit (4)**	**Very Much (5)**
Shortness of breath	351 (90.7)	36 (9.3)	3 (0.78)	26 (6.72)	49 (12.66)	**87 (22.48)**	**186 (48.06)**
Pain	307 (79.33)	80 (20.67)	4 (1.03)	33 (8.53)	54 (13.95)	**83 (21.45)**	**132 (34.11)**
Cough (1 missing)	300 (77.52)	86 (22.22)	13 (3.36)	41 (10.59)	53 (13.70)	**69 (17.83)**	**127 (32.82)**
Feeling drowsy	285 (73.64)	102 (26.36)	6 (1.55)	41 (10.59)	74 (19.12)	**90 (23.26)**	**74 (19.12)**
Lack of energy (2 missing)	280 (72.35)	105 (27.13)	11 (2.84)	46 (11.89)	57 (14.73)	**57 (14.73)**	**107 (27.65)**
Difficulty sleep (1 missing)	263 (67.96)	123 (31.78)	4 (1.03)	19 (4.91)	37 (9.56)	**67 (17.31)**	**136 (35.14)**
Dry mouth	216 (55.81)	171 (44.19)	10 (2.58)	31 (8.01)	53 (13.7)	**51 (13.18)**	**71 (18.35)**
Numbness (tingling in hands/feet) (1 missing)	204 (52.71)	182 (47.03)	4 (1.03)	27 (6.98)	59 (15.15)	59 (15.25)	55 (14.21)
Difficulty concentrating (1 missing)	195 (50.39)	191 (49.35)	16 (4.13)	17 (4.39)	47 (12.14)	57 (14.73)	57 (14.73)
Weight loss (6 missing data)	180 (46.51)	201 (51.94)	13 (3.36)	35 (9.04)	46 (11.89)	57 (14.73)	28 (7.24)
Dizziness	175 (45.22)	212 (54.78)	6 (1.55)	52 (13.44)	52 (13.44)	42 (10.85)	25 (6.46)
Nausea	165 (42.64)	222 (57.36)	3 (0.78)	33 (8.53)	46 (11.89)	48 (12.4)	35 (9.04)
Sweats (2 missing data)	164 (42.38)	221 (57.11)	16 (4.13)	28 (7.24)	42 (10.85)	42 (10.85)	36 (9.3)
Itching (1 missing)	144 (37.31)	242 (62.53)	4 (1.03)	21 (5.43)	39 (10.08)	44 (11.37)	35 (9.04)
Feeling bloated (1 missing)	137 (35.4)	249 (64.34)	6 (1.55)	23 (5.94)	34 (8.79)	33 (8.53	39 (10.08)
Lack of appetite (1 missing data)	134 (34.63)	252 (65.12)	6 (1.55)	22 (5.68)	27 (6.98)	38 (9.82)	42 (10.85)
Changes in skin (3 missing data)	130 (33.59)	254 (65.63)	11 (2.84)	16 (4.13)	25 (6.46)	38 (9.82)	40 (10.34)
I do not like myself	130 (33.59)	257 (66.41)	5 (1.29)	40 (10.34)	28 (7.24)	28 (7.24)	30 (7.75)
Swelling of arms/legs	124 (32.04)	263 (67.96)	1 (0.26)	24 (6.2)	29 (7.49)	46 (11.89)	25 (6.46)
Constipation (1 missing)	119 (30.75)	267 (68.99)	2 (0.52)	28 (7.24)	27 (6.98)	25 (6.46)	38 (9.82)
Urinating problems	92 (23.77)	295 (76.23)	2 (0.52)	15 (3.88)	27 (6.98)	25 (6.46)	22 (5.68)
Changes in food taste	84 (21.71)	303 (78.29)	2 (0.52)	22 (5.68)	26 (6.72)	18 (4.65)	16 (4.13)
Problems with sexual activity (7 missing data)	80 (20.67)	300 (77.52)	8 (2.07)	10 (2.58)	12 (3.1)	21 (5.43)	27 (6.98)
Vomiting	58 (14.99)	329 (85.01)	2 (0.52)	12 (3.1)	24 (6.2)	11 (2.84)	8 (2.07)
Difficulty swallowing (2 missing)	56 (14.47)	329 (85.01)	1 (0.26)	19 (4.91)	15 (3.88)	14 (3.62)	7 (1.81)
Diarrhoea (3 missing data)	55 (14.2)	329 (85.01)	2 (0.52)	16 (4.13)	16 (4.13)	8 (2.07)	11 (2.84)
Hair loss (2 missing)	33 (8.53)	352 (90.96)	4 (1.03)	7 (1.81)	7 (1.81)	8 (2.07)	6 (1.55)
Mouth sores	20 (5.17)	367 (94.83)	2 (0.52)	6 (1.55)	7 (1.81)	4 (1.03)	1 (0.26)
**Psychological Symptoms**	**Prevalence**	**Not Present**	**Burden of Prevalent Symptoms n (%)**				
			**Rarely**	**Occasionally**	**Frequently**	**Almost Constantly**	
Worrying (2 missing)	252 (65.12)	133 (34.37)	13 (3.36)	65 (16.8)	**81 (20.9)**	**93 (24.03)**	
Feeling sad	232 (59.95)	155 (40.05)	7 (1.81)	71 (18.35)	**77 (19.9)**	**77 (19.9)**	
Feeling irritable (2 missing data)	200 (51.68)	185 (47.8)	11 (2.84)	71 (18.35)	**62 (16.02)**	**55 (14.21)**	
Feeling nervous (1 missing data)	157 (40.57)	229 (59.17)	7 (1.81)	54 (13.95)	53 (13.7)	42 (10.85)	

**Notes**: The most burden-some physical symptoms (quite a bit and very much) and psychological symptoms (frequently and almost constantly) have been highlighted with bold text.

Total scores were calculated for MOS-SSS, LCADL and CAT.

MSAS-GDI, MSAS-PHYS and MSAS-PSYCH scores were converted into quartiles because they were not normally distributed. The KPS scores were skewed, we therefore divided into two groups: (1) score of ≤70% (Cares for self; unable to carry on normal activity or do work), (2) score of ≥80% (ability to carry normal activity and with effort; some signs or symptoms of disease). Research has recommended the use an algorithm with a minimum of two and a maximum of three variables to facilitate an adequate and efficient evaluation of the KPS.^47^ We used descriptive statistics to profile demographic, socioeconomic and clinical characteristics of the sample.

Global distress index (MSAS-GDI), physical symptom distress (MSAS-PHYS) and psychological symptom distress (MSAS-PSYCH) were dependent outcomes. Demographic variables (age, sex, education, smoking status, living situation, living arrangement), clinical variables (COPD Gold stage, treatment adherence, diagnosis, and KPS) were covariates. Other covariates were number of missed-dose of prescribed medication, The COPD Assessment Test (CAT) scores, Severity of breathlessness on exertion (the London Chest Activity of Daily Living Questionnaire) and social support (MOS-SSS). The first step involved conducting a univariate ordinal logistic regression analysis for all demographic, and clinical variables. Second step involved constructing multivariate ordinal logistic regression models for all variables falling within less than 25% p value^48^ as shown on Tables 3–5. We performed regression analysis for all the three models adjusting for all the predictor values. The mean VIF (variance inflation factor), a measure of the amount of multicollinearity in a set of multiple regression variables was 1.23. As a rule of thumb, VIF values less than 10 indicate no multicollinearity between the variables.^49^

**Table 3:** Ordinal Logistic Regression Model: Association of Global Distress Index (MSAS-GDI) with Social Support, Activities of Living, COPD Score, Demographic, and Clinical Factors (n=380)

Independent Variables	Univariate Analysis		Multivariate Analysis	
	Odds Ratio (95% CI)	P value	Odds Ratio (95% CI)	P value
Age	0.96 (0.94 to 0.98)	<0.001*	0.97 (0.95 to 0.99)	0.004*
Men vs women (ref: male)	1.3 (.9 to 1.85)	0.16	1.21 (0.81 to 1.81)	0.36
KPS (in two groups): ≤70% vs ≥ 80% score	0.54 (0.38 to 0.78)	0.001*	1.16 (0.74 to 1.81)	0.52
Missed 1 or more doses (ref No)	2.64 (1.62 to 4.29)	<0.001*	2.05 (1.23 to 3.43)	0.006*
Co-morbidity: (None vs ≥ 1 co-morbidity)	2.39 (1.55 to 3.69)	<0.001*	1.35 (0.83 to 2.21)	0.22
Smoking status (ref yes)	0.78 (0.54 to 1.12)	0.18	0.93 (0.63 to 1.39)	0.74
Living arrangement: (lives alone vs does not live alone)	1.25 (0.68 to 2.31)	0.47	–	–
Living situation: formal with sanitation inside vs formal/informal with sanitation outside	1.40 (0.88 to 2.21)	0.15	0.87 (0.52 to 1.46)	0.61
Hospitalisations in the last 12 months (None vs ≥ 1)	1.38 (0.96 to 1.99)	0.08	0.95 (0.62 to 1.47)	0.82
Education (Primary vs high school and beyond)	0.83 (0.58 to 1.18)	0.30	–	–
Income: employed vs unemployed	1.1 (0.57 to 2.16)	0.77	–	–
Employed vs disability grant/pension	0.76 (0.43 to 1.34)	0.34		
COPD Assessment Test (CAT)	1.14 (1.1 to 1.17)	<0.001*	1.09 (1.06 to 1.13)	<0.001*
Activity of daily living (LCADL)	1.08 (1.06 to 1.1)	<0.001*	1.05 (1.03 to 1.07)	<0.001*
Social support (MOS-Social Support Survey)	0.98 (.97 to 0.99)	<0.001*	0.98 (0.97 to 0.99)	<0.001*

**Note**: *Significant at <0.05.

**Abbreviations**: KPS, Karnofsky Performance Status; LCADL, London Chest Activity of Daily Living Scale; MOS, Medical Outcomes Study.

**Table 4:** Ordinal Logistic Regression Model: Association of Psychological Symptom Distress Index (MSAS-PSYCH) with Social Support, Activities of Living, COPD Score, Demographic, and Clinical Factors (n=377)

Independent Variables	Univariate Analysis		Multivariate Analysis	
	Odds Ratio (95% CI)	P value	Odds Ratio (95% CI)	P value
Age	0.97 (0.95 to 0.98)	<0.001*	0.98 (0.95 to 0.99)	0.032*
Men vs women (ref male)	1.23 (0.86 to 1.76)	0.25	1.31 (0.88 to 1.96)	0.19
KPS (in two groups): ≤70% vs ≥ 80% score	0.59 (0.41 to 0.85)	0.005*	1.38 (0.87 to 2.19)	0.17
Missed 1 or more doses (ref No)	1.64 (1.01 to 2.65)	0.045*	1.30 (0.78 to 2.18)	0.32
Co-morbidity: (None vs ≥ 1 co-morbidity)	2.06 (1.34 to 3.18)	<0.001*	1.09 (0.66 to 1.78)	0.75
Smoking status (ref yes)	0.80 (0.56 to 1.15)	0.23	0.93 (0.62 to 1.38)	0.71
Living arrangement: alone vs does not live alone	1.37 (0.73 to 2.54)	0.32	–	
Living situation: formal with sanitation inside vs formal/informal with sanitation outside	1.60 (1.01 to 2.53)	0.046*	1.18 (0.70 to 1.98)	0.53
Hospitalisations in the last 12 months (None vs ≥ 1)	1.45 (1.01 to 2.09)	0.045*	1.15 (0.75 TO 1.77)	0.53
Education (Primary vs high school and beyond)	0.88 (0.62 to 1.26)	0.49	–	–
Income: employed vs unemployed	1.51 (0.75 to 3.02)	0.25	1.08 (0.84 to 3.78)	0.13
Employed vs disability grant/pension	0.96 (0.53 to 1.73)	0.88	1.45 (0.72 to 2.90)	0.29
COPD Assessment Test (CAT)	1.13 (1.1 to 1.16)	<0.001*	1.08 (1.04 to 1.11)	<0.001*
Activity of daily living (LCADL)	1.07 (1.05 to 1.08)	<0.001*	1.05 (1.03 to 1.07)	<0.001*
Social support (MOS-Social Support Survey)	0.98 (0.96 to 0.99)	<0.001*	0.97 (0.96 to 0.98)	<0.001*

**Note**: *Significant at <0.05.

**Abbreviations**: KPS, Karnofsky Performance Status; LCADL, London Chest Activity of Daily Living Scale; MOS, Medical Outcomes Study.

**Table 5:** Ordinal Logistic Regression Model: Association of Physical Symptom Distress Index (MSAS-PHYS) with Social Support, Activities of Living, COPD Score, Demographic, and Clinical Factors (n=379)

Independent Variables	Univariate Analysis		Multivariate Analysis	
	Odds Ratio (95% CI)	P value	Odds Ratio (95% CI)	P value
Age	0.96 (0.95 to 0.98)	<0.001*	0.98 (0.95 to 0.99).	0.030*
Men vs women (ref male)	1.37 (0.96 to 1.96)	0.09	1.41 (0.95 to 2.08)	0.09
KPS (in two groups): ≤70% vs ≥ 80% score	0.61 (0.43 to 0.88)	0.008*	1.15 (0.75 to 1.76)	0.53
Missed 1 or more doses (ref No)	2.34 (1.44 to 3.81)	0.001*	2.10 (1.26 to 3.51)	0.004*
Co-morbidity: (None vs ≥ 1 co-morbidity)	2.95 (1.89 to 4.59)	<0.001*	1.75 (1.09 to 2.83)	0.021*
Smoking status (ref yes)	0.95 (0.67 to 1.37)	0.80	–	–
Living arrangement: alone vs does not live alone	1.21 (0.66 to 2.22)	0.53	–	–
Living situation: formal with sanitation inside vs formal/informal with sanitation outside	1.18 (0.75 to 1.85)	0.48	–	–
Hospitalisations in the last 12 months (None vs ≥ 1)	1.17 (0.81 to 1.69)	0.39	–	–
Education (Primary vs high school and beyond)	0.85 (0.59 to 1.21)	0.35	–	–
Income: employed vs unemployed	0.87 (0.44 to 1.72)	0.69	1.06 (0.51 to 2.23)	0.87
Employed vs disability grant/pension	0.70 (0.39 to 1.25)	0.23	0.92 (0.46 to 1.83)	0.81
COPD Assessment Test (CAT)	1.15 (1.12 to 1.19)	<0.001*	1.11 (1.07 to 1.15)	<0.001*
Activity of daily living (LCADL)	1.08 (1.06 to 1.1)	<0.001*	1.04 (1.02 to 1.06)	<0.001*
Social support (MOS-Social Support Survey)	0.99 (0.98 to 0.99)	0.005*	0.99 (0.98 to 1.00)	0.08

**Note**: *Significant at <0.05.

**Abbreviations**: KPS, Karnofsky Performance Status; LCADL, London Chest Activity of Daily Living Scale; MOS, Medical Outcomes Study.

## Results

### Participation and Sample Characteristics

Table 1 presents the demographic and clinical characteristics of the sample. Of 469 participants approached, 54 declined to participate, 28 were excluded (mainly due to inaccurate file recording of diagnosis, and inability to consent) and therefore 387 participated. The response rate was 82.5%. The mean age was 59.5 years (SD 10.1), range 25–89 years. Just over half were females (n=205, 53.0%). Over half of the sample (n=213, 55.0%) were Afrikaans, and just less than half (n=190, 49.1%) attended primary school without attending high school. Nearly half of the sample (n=174 45.19%) were smokers at the time of interview.

### Treatment Variables

Treatment variables are presented in Table 1. The majority of patient’s records did not have COPD GOLD staging (n=337). Of the n=50 patients who had COPD staging recorded n=29; 58% were at stage 2. Adherence to treatment was high with n=318 (82.6%) participants reporting that they did not miss any dose for the last month. Just over half of the sample had one or more hospitalisations (n=199; 53.07%) in the previous 12 months.

One-filth of the sample was referred for respiratory medicine in the last 12 months (n=73; 18.86%). The majority of the patients did not have a comorbidity (n=298; 77%). Nearly half of the sample had a KPS score of ≤70% (n=184; 47.92%).

### Symptoms Reported

The ten most prevalent symptoms are presented in Table 2. Shortness of breath was the most prevalent symptom (n=351, 90.7%), followed by pain (n=307, 79.33%), cough (n=300, 77.72%), feeling drowsy (n=285, 73.64%), lack of energy (n=280, 72.73%), sleeping difficulties (n=263, 68.13%), worry (n=252, 65.45%), feeling sad (n=232, 9.95%), dry mouth (n=216, 55.81%), and numbness (n=204, 52.85%).

The most burdensome physical symptoms (worst two categories) reported as “quite a bit” or “very much” and psychological symptoms “frequently” or “almost constantly” were shortness of breath (70.54%), pain (55.56%), difficulty sleeping (52.45%), cough (50.65%), worry (44.93%), feeling drowsy (42.38%), lack of energy (42.38%), feeling sad (39.8%), dry mouth (31.53%), and feeling irritable (30.23%). The mean symptom distress indices for the three subscales were 1) global distress index (GDI) 16.69 (SD=9.12), 2) physical symptom distress (PHYS) 15.86 (SD=9.56) and 3) psychological symptom distress (PSYCH) was 10.05 (SD=6.39).

### Predictors of Symptom Prevalence and Distress

Univariate and multivariate ordinal logistic regression models are presented in Tables 3–5.

Table 3 shows that in univariate analysis global distress was associated with age, KPS score, missing 1 or more doses, co-morbidity, COPD Assessment Test (CAT) (health status impairment or impact of COPD on a person’s life), activities of daily living (severity of breathless on exertion) and social support.

In a multivariate analysis, global symptom distress was positively associated with CAT scores (COPD impairment on person’s life) odds ratio 1.09; 95% CI 1.06 to 1.13; p<0.001, and poorer ability to perform activities of daily living (odds ratio 1.05; 95% CI 1.03 to 1.07 P<0.001). Global symptom distress was also associated with low social support (odds ratio 0.98; 95% CI 0.97 to 0.99; p<0.001). Old age was the predictor for lower (better) global symptom distress (odds ratio 0.97; 95% CI 0.95 to 0.99; P=0.004). Missing 1 or more doses of prescribed medication was associated with increasing levels of global symptom distress (odds ratio 2.05; 95% CI 1.23 to 3.43; p=0.006).

In Table 4, univariate analysis shows that psychological symptom distress was significantly associated with age, KPS score, number of missed doses of prescribed medication, co-morbidity, living situation, hospitalisation in the last 12 months, COPD Assessment Test (CAT), activities of daily living and social support.

In multivariate analysis, every unit increase with age (in years) was associated with better (lower) psychological distress status (odds ratio 0.98; 95% CI 0.95 to 0.99; P=0.032). Psychological symptom distress was positively associated with COPD impairment (CAT score) (odds ratio 1.08; 95% CI 1.04 to 1.11; P<0.001) and inability to perform activities of daily living (odds ratio 1.05; 95% CI 1.03 to 1.07; p<0.001). Furthermore, psychological distress was associated with low social support (odds ratio 0.97; 95% CI 0.96 to 0.98; p<0.001).

In Table 5 univariate analysis shows that old age was predictor of low (better) physical symptom distress, having a higher KPS was associated with better (lower) physical symptom distress. Missing 1 or more doses of prescribed medication and having a co-morbidity was associated with worsening physical distress. COPD Assessment Test (CAT), social support and activities of daily living were all associated with physical distress.

Multivariate analysis shows that physical symptom distress was associated with health status of the individual’s (worse CAT scores) (odds ratio 1.11; 95% CI; 1.07 to 1.15; P<0.001) inability to perform activities of daily living (odds ratio 1.04; 95% 1.02 to 1.06; P<0.001). Age was a predictor for lower (better) physical symptom distress (odds ratio 0.98; 95% CI 0.95 to 0.99; p=0.030). Missing 1 or more doses of prescribed medication was associated with increasing levels/scores of physical distress (odds ratio 2.1 95% CI 1.26 to 3.51; P=0.004). Physical symptom distress was worse among participants who had one or more co-morbidity (odds ratio 1.75 95% CI 1.09 to 2.83; P=0.021).

## Discussion

### Summary of Study Findings

Our study has revealed a high prevalence and burden of both physical and psychological symptoms among chronic lung disease patients attending primary care in South Africa.

The ten most burdensome symptoms (the worst two categories) of physical symptoms “quite a bit” or “very much” and psychological symptoms “frequently” or “almost constantly” were shortness of breath (70.54%), pain (55.56%), difficulty sleeping (52.45%), cough (50.65%), worry (44.93%), feeling drowsy (42.38%), lack of energy (42.38%), feeling sad (39.8%), dry mouth (31.53%), and feeling irritable (30.23%).

The high prevalence and burden of shortness of breath among COPD patients has been reported in a systematic review with prevalence range from 45% to 60%.^50^ The high prevalence and burden of psychological symptoms such as worry, sadness and feeling irritable may be attributed to the physical symptoms of pain, shortness of breath and cough which makes patients to worry and feel sad about their illness. Lack of sleep maybe be attributed to shortness of breath and worry which eventually can lead to the symptom of feeling drowsy during the day.

Higher global symptom distress was positively/significantly associated with worse CAT scores (impairment on the participant’s wellbeing and daily life), poorer ability to perform activities of daily living and poor social support. Symptom burden can affect patients’ ability to perform activities of living such as working, walking, bathing.^50^,^51^

This has an impact on the quality of life of COPD patients. Research has shown that poor quality of life is associated with significant increases in COPD respiratory symptoms (dyspnoea/shortness of breath, cough, and expectoration).^52^

Both physical and psychological symptom distress were also significantly associated with patient’s wellbeing and daily life (worse CAT scores) and inability to perform activities of daily living. Participants who reported a higher/worse psychological symptom distress were significantly associated with poor social support. Social support is very important for mental wellbeing of the patients. This includes family, friends and community.^53^

A study in a HIC setting reported that lack of help from others leads to anxiety and depression among COPD patients,^54^ while in another HIC setting higher patient recognised social support has been associated with better COPD outcomes such as physical and psychological symptoms and quality of life.^55^ A narrative review to understand the impact of symptoms on the burden of COPD using real-world data from Europe, cross-sectional and observational studies concluded that symptom distress had significant negative effects on patients’ ability to perform normal physical activities throughout the day.^50^ A longitudinal observational study conducted in the USA reported that symptom distress (MSAS-GDI) was associated with impaired quality of life, functional impairment, female sex and poor psychological well-being.^56^ These findings are similar to our study except the gender variable.

Participants may have missed 1 or more doses because of the effects of both global distress, physical and psychological distress.

The mean (SD) CAT score was 26.39 (7.57). This is considered to have a high impact on patient’s wellbeing and daily life. Anything greater than 20 is considered high impact.^41^ This can be attributed to high prevalence and burden of dyspnoea, pain, insomnia and related symptoms. This possibly explains why the patients were distressed. Only n=50 patients of the whole sample were staged. The South African COPD guidelines recommend that every patient should be staged.^6^ However, in primary care in South Africa, the diagnosis of COPD is typically clinical and considers a wide range of aetiologies. This is as per local recommended guidelines: In Cape Town, the Western Cape Department of Health PACK Guidelines (https://knowledgetranslation.co.za/pack/wc-south-africa/) are used. PACK guidelines have been endorsed for a symptom-based approach to primary care in South Africa.^57^ PACK provides guidelines for the diagnosis and management of COPD based on symptoms and peak expiratory flow rate, with recommendation for referral for spirometry if available. However, spirometry is usually not available in the primary care setting and requires travel to tertiary centres, posing a barrier to spirometry for these patients. There is a need therefore to address the impact of the limited access to spirometry for primary care facilities. Improved access will ensure that patients are thoroughly assessed, and clinicians will make proper treatment decisions since this is critical for detection, assessment and management of patients with COPD.^6^

The mean symptom distress indices for the three subscales were 1) global distress index (GDI) 16.69 (SD=9.12), 2) physical symptom distress (PHYS) 15.86 (SD=9.56) and 3) psychological symptom distress (PSYCH) was 10.05 (SD=6.39). These are higher compared to our previous work in HIV population GDI mean (SD) 13.34 (10.06), physical symptom distress 12.52 (9.88) and psychological symptom distress 8.44 (7.22),^58^ and cancer population GDI mean (SD) 1.61 (SD = 0.70), physical distress 1.41 (SD = 0.75) and the psychological distress 1.33 (SD = 0.76).^35^ These data carry important implications for clinical care and services. The prevalence of multidimensional symptoms underlines the importance of person-centred care approaches to assessment and management of patient problems and concerns, taking account of multiple and potentially interacting symptoms and locally appropriate resources to reduce distress.

Adherence to treatment was 83% in this sample. The high adherence to therapeutic strategies is due to public health interventions that have been put in place in South Africa to support people to adhere to therapy. For example, in HIV primary care settings in South Africa adherence rate was at 95%^59^ and 71% in heart failure.^60^

This is the first study to be conducted in South Africa in primary care setting across eight facilities with different models of care. This work reveals the problems among COPD patients in primary care and informs policy on the needs for improvement in the delivery of services in primary care in South Africa.

### Strengths and Limitations

We had a large sample size in our study (n=387), with a high response rate (82%). Our research assistants were local researchers who understand the language and the culture of study participants. The research questionnaires were administered by the research assistants. The research assistant read aloud questionnaires to each participant and recorded participants’ responses. We believe this strategy enabled us to achieve the high response rate and low missing data. We have used the same strategy in HIV populations in Kenya^58^,^61^ and we recorded a response rate of 84%.

This study was cross-sectional; therefore, we cannot determine casual associations, and we acknowledge a potential sampling bias in that those patients unable to travel to clinic were not approached in our recruitment procedures.

An important finding of our study was that only n=50/387 participants had COPD staging on file. This study was conducted in primary care where diagnosis is made clinically. We extracted this data from patients’ records/files, reflecting current practice. Lack of COPD staging in our study must inform clinical policy development in South Africa.

A previous study on “Primary care physician perceptions on the diagnosis and management of chronic obstructive pulmonary disease in diverse regions of the world” reported that spirometry was not used. The authors concluded that “that COPD guidelines appear to have limited reach and applications in these areas”.^62^ In the absence of spirometry, a recent multi-national low- and middle-income countries study found that screening tools are feasible to administer (and that 95% of those with COPD were previously unaware of the diagnosis despite high prevalence of clinically important symptoms and lower quality of life).^63^

Our study reflects the realities of conducting research in LMICs and can inform primary care practice.

Only 49% of the sample reached primary education. These findings are reflections of the population. South Africa faces an obstacle in the education system due to school drop-outs among youth.^64^ Even though MSAS had been validated in cancer and heart failure populations in sub-Saharan Africa,^32^,^33^ the tool has not been validated in COPD population in South Africa.

### Recommendations/Research Implications

Evidence from high-income countries has shown that an integrated palliative care service for patients with serious health-related suffering experiencing breathlessness^65^ (with 54% of the sample having a diagnosis of COPD) has positive benefits on mastery of breathlessness, survival, and cost savings.^66^ The intervention included a self-management component, which offers great potential for this community-dwelling population. A strong body of evidence from several systematic reviews has shown that self-management reduces breathlessness and hospitalisation, and improves quality of life and distress, satisfaction, and survival.^67–70^ Given the high symptom prevalence and burden, and the association between breathlessness in COPD and poor 5-year survival,^71^ COPD patients must benefit from the new Universal Health Coverage goals, for accessible, affordable and streamlined access to accurate diagnoses and importantly including palliative care as an essential health service.

Our work informs clinical practice on the need for patient education around physical and psychological interventions and integration of palliative care within multidisciplinary outpatient respiratory services to address the current challenges.

A systematic review of palliative care and management of troublesome symptoms in COPD concluded that early integration of palliative care in across all levels of care including primary care with respiratory, rehabilitation and referral services based on complexity of physical and psychological symptoms rather than prognosis can improve patient outcomes. These models of integration include management of refractory breathlessness, short-term palliation services in facilities with limited access.^69^ Pulmonary rehabilitation services such as self-management education and exercises improves patients ability to tolerate exercises, breathlessness, health status, and psychological morbidity.^72^

## Conclusion

The high prevalence and burden of physical and psychological symptoms provides strong evidence of the need for integrating person-centred assessment and management of symptoms in primary care settings for chronic lung disease patients. This also underlines the importance of person-centred care assessment incorporating multiple interacting physical and psychological symptoms and concerns and management using locally appropriate resources.
